# Renal Function Recovery Strategies Following Marathon in Amateur Runners

**DOI:** 10.3389/fphys.2022.812237

**Published:** 2022-02-28

**Authors:** Carlos Hernando, Carla Hernando, Nayara Panizo, Eladio Collado-Boira, Ana Folch-Ayora, Ignacio Martínez-Navarro, Barbara Hernando

**Affiliations:** ^1^Sport Service, Jaume I University, Castellon, Spain; ^2^Department of Education and Specific Didactics, Jaume I University, Castellon, Spain; ^3^Department of Mathematics, Carlos III University of Madrid, Madrid, Spain; ^4^Nephrology Service, University Clinical Hospital of Valencia, Valencia, Spain; ^5^Faculty of Health Sciences, Jaume I University, Castellon, Spain; ^6^Department of Physical Education and Sport, University of Valencia, Valencia, Spain; ^7^Sports Health Unit, Vithas-Nisa 9 de Octubre Hospital, Valencia, Spain; ^8^Department of Medicine, Jaume I University, Castellon, Spain

**Keywords:** acute kidney injury, marathon, glomerular filtration rate, active recovery, passive recovery

## Abstract

Long distance races have a physiological impact on runners. Up to now, studies analyzing these physiological repercussions have been mainly focused on muscle and cardiac damage, as well as on its recovery. Therefore, a limited number of studies have been done to explore acute kidney failure and recovery after performing extreme exercises. Here, we monitored renal function in 76 marathon finishers (14 females) from the day before participating in a marathon until 192 h after crossing the finish line (FL). Renal function was evaluated by measuring serum creatinine (sCr) and the glomerular filtration rate (GFR). We randomly grouped our cohort into three intervention groups to compare three different strategies for marathon recovery: total rest (REST), continuous running at their ventilatory threshold 1 (VT1) intensity (RUN), and elliptical workout at their VT1 intensity (ELLIPTICAL). Interventions in the RUN and ELLIPTICAL groups were performed at 48, 96, and 144 h after marathon running. Seven blood samples (at the day before the marathon, at the FL, and at 24, 48, 96, 144, and 192 h post-marathon) and three urine samples (at the day before the marathon, at the finish line, and at 48 h post-marathon) were collected per participant. Both heart rate monitors and triaxial accelerometers were used to control the intensity effort during both the marathon race and the recovery period. Contrary to our expectations, the use of elliptical machines for marathon recovery delays renal function recovery. Specifically, the ELLIPTICAL group showed a significantly lower ∆GFR compared to both the RUN group (*p* = 4.5 × 10^−4^) and the REST group (*p* = 0.003). Hence, we encourage runners to carry out an active recovery based on light-intensity continuous running from 48 h after finishing the marathon. In addition, full resting seems to be a better strategy than performing elliptical workouts.

## Introduction

Given the increase of marathon running popularity, the physiological alterations caused by performing such a demanding effort have increased the interest of the scientific community (Scheer, 2019; Rojas-Valverde et al., 2021b; Scheer et al., 2021). Running a long-distance race demands a vigorous physical effort, which has been shown to generate transient elevation of biomarkers associated with pathological conditions such as muscle damage, inflammation, heart damage, and renal failure (Briviba et al., 2005; Khodaee et al., 2015; Belli et al., 2018; Knechtle and Nikolaidis, 2018; Nikolaidis et al., 2018; Bernat-Adell et al., 2019, 2020; Martínez-Navarro et al., 2020a,c; Scheer et al., 2021).

In the last few years, several studies have focused on studying acute kidney injury (AKI) after performing a physically demanding exercise (Lipman et al., 2014; Traiperm et al., 2016; Mansour et al., 2017; González et al., 2019; Rojas-Valverde et al., 2019; Poussel et al., 2020; Khodaee et al., 2021). Contrary to other acute pathological alterations, renal function has been shown to be normalized 24 h after running a long-distance race. As a result, the collection of biomarkers related to AKI have been usually restricted to the running phase (Lipman et al., 2014; Belli et al., 2018) or to the first 24 h of the recovery phase (McCullough et al., 2011; Traiperm et al., 2016; Mansour et al., 2017; Khodaee et al., 2021). However, we observed that glomerular filtration rate (GFR) significantly worsened 48 h after marathon running (González et al., 2019). Therefore, there is a need for monitoring renal function more than 48 h after performing a strenuous exercise.

The recovery of exercise-associated physiological damage has been a matter of concern for sport science researchers, coaches, and medical staff. Recent studies aimed at determining how long is needed, as well as what is the best strategy, for recovering from muscle damage and for normalizing neuromuscular performance after performing a long-distance race (Sherman et al., 1984; Wiewelhove et al., 2018; Martínez-Navarro et al., 2020b). These studies analyzed the effect of different recovery strategies usually followed by marathoners (massage, cold water immersion, total rest, light running, and elliptical machine workouts) on muscle damage recovery without obtaining any conclusive results. However, a study that comprehensively characterizes renal function normalization including a significant cohort of runners is lacking in the field.

Physical exercise increases body temperature and leads to peripheral vasodilatation and blood flow (Poortmans, 1984; Poortmans et al., 1988; Ferreira et al., 2019). This fact promotes the activation of renin-angiotensin-aldosterone system, which increases the filtration pressure and consequently the GFR. Given that physical exercise has shown a beneficial effect in patients with chronic kidney disease (Johansen and Painter, 2012; Heiwe and Jacobson, 2014; Viana et al., 2014; Greenwood et al., 2015; Santana et al., 2017), we hypothesized that performing a low-impact physical activity will accelerate the recovery of marathon-induced acute kidney damage faster than resting during the whole post-marathon week.

Here, we present a research study focused on exploring the effects of exercise within a proposed strategy to optimize kidney function recovery following the completion of a marathon race. Our large cohort of 76 marathon finishers allowed us to compare the effect of three different recovery strategies (resting, running, and elliptical workout) on renal function after a marathon race.

## Materials and Methods

### Sample Set and Subsampling

All participants of the Valencia Fundación Trinidad Alfonso EDP 2016 Marathon received an invitation by email to participate in this study. Three informative seminars were organized to fully explain the study design to those individuals who accepted the invitation (*n* = 456). A total of 98 recreational marathon runners were selected to participate in this study, according to the following inclusion criteria: (1) being between 30 and 45 years old; (2) having a previous marathon experience, with a marathon personal best between 3 and 4 h for males and between 3:30 and 4:30 h for females; (3) having a body mass index (BMI) between 16 and 24.99; and (4) being free from cardiovascular disease, renal dysfunction, and dyslipidemia. All individuals selected were fully informed and gave their written consent to participate. The research was conducted according to the Declaration of Helsinki, and it was approved by the Research Ethics Committee of the Jaume I University of Castellon. This work is part of a project aiming at finding the best strategy to recover from marathon-induced physiological damage. This project is enrolled in the https://clinicaltrials.gov/ct2/show/NTC03155633 database, with the code number NCT03155633.^1^ Therefore, the same study population was used in previous publications (Martínez-Navarro et al., 2020a,b,c).

Ninety-five out of 98 volunteers started the Valencia Marathon on 22 November 2016. From them, 88 (74 males and 14 females) crossed the finish line (FL) and were thus randomly included in the three intervention groups, which were monitored until 192 h after crossing the FL. Although 78 runners completed the entire study, two of them were discarded from the analysis because they consumed non-steroidal anti-inflammatory drugs (NSAIDs) during the recovery phase. Therefore, a total of 76 runners were finally used to explore the hypotheses of this study.

Each group of runners followed one of the three strategies to test during the recovery phase. The first group (*N* = 32; six females) did not perform any physical activity (REST group). The second group (*N* = 22; four females) performed a 40-min run (RUN group). The third group (*N* = 22; four females) performed a 40-min workout on an elliptical machine (Synchro excite 500, Technogym, Cesena, Italia; ELLIPTICAL group). All participants who used the elliptical machine in the recovery period had previously used this tool, although it was not their usual training method. In any case, we allowed runners to get used to the elliptical machine during 5 min before each session of the recovery phase. Since there are biomechanical differences between the three possible positions (using the handles, holding onto a central bar and free-hand) of using an elliptical machine (Jackson et al., 2010; Moreside and McGill, 2012), participants were not allowed to perform workout sessions holding the central bar.

We selected three biomechanically similar activities because: (1) all three activities generate equivalent movements in the Cartesian coordinate axes allowing to have comparable accelerometry-based estimation of energy expenditure (Cordero et al., 2014; Rowlands et al., 2014; de Almeida Mendes et al., 2018; Hernando et al., 2018, 2020), (2) the intervention of gravity is not significantly disturbed when performing the activity movement (unlike in cycling or swimming), (3) the activity movements are similar to those performed in marathon training sessions and racing, and (4) participants can be continuously controlled by researchers when doing the two active strategies.

Runners performed three times the same workout in the recovery phase (at 48, 96, and 144 h after finishing the marathon). The physical intensity required was between 95 and 105% of their ventilatory threshold 1 (VT1). To control the physical intensity at which runners were performing the physical activity, each runner wore a heart rate monitor (Polar M400 HR monitor, Kempele, Finland). All interventions were supervised by experts to guarantee that workouts were correctly performed by runners. Although preventive strategies can be applied for accelerating recovery from marathon-related AKI (Juett et al., 2020), no intervention was performed to prevent AKI. No participant expressed physical limitations for performing workouts during the recovery period. No control of food and liquid intake was performed during the whole study.

In addition, the physical activity done by each runner throughout the study was monitored using accelerometer devices. Each participant wore a GENEActiv accelerometer (Activinsights Ltd., Kimbolton, Cambridgeshire, United Kingdom) on the non-dominant wrist as a watch from the day before the marathon until 192 h post-race. Applying our validated approach (Hernando et al., 2018), we calculated the relative caloric consumption (kcal·kg^−1^·min^−1^) every 8 h following the circadian rhythm criteria (Vitale et al., 2015). For comparison of the relative caloric consumption, we added the caloric consumption of three different moments: (1) the time elapsed from arrival at finish line to 48 h post-race (six 8-h segments), (2) the three 8-h segments where the activity was performed (24 h in total), and (3) the time of the intervention phase where runners are not performing the activity (13 8-h segments).

### Data Collection

#### Training- and Competition-Related Data

A suitable questionnaire (Hernando et al., 2018) was used to collect demographic, sociographic, and medical information, the training plan, and competition history.

#### Cardiopulmonary Exercise Test

Prior to running the marathon, all individuals selected for this study performed a cardiopulmonary exercise test on a treadmill (pulsar® 3p, h/p/cosmos Sports and Medical GmbH, Nussdorf-Traunstein, Germany) until exhaustion. Breath-by-breath gas exchange was measured by the Jaeger MasterScreen® CPX gas analyzer to identify the first ventilatory threshold (VT1), the second ventilatory threshold (VT2), and the maximal oxygen consumption (V·O_2max_; Skinner et al., 1980; McLellan and Skinner, 1985).

#### Blood Samples

Seven blood samples (at the day before the marathon, at the finish line, and at 24, 48, 96, 144, and 192 h post-marathon) were collected per participant. These samples were taken from runners’ antecubital veins by venipuncture using BD Vacutainer PST II tubes, centrifuged at 3,500 rpm for 10 min, and transported to the Vithas NISA Hospital in Valencia at 4°C for biochemical analysis. In the recovery phase, blood samples were collected prior to performing the active recovery workouts. The impact of dehydration on plasma volume alterations was taken into account in the analysis of biochemical parameters collected at post-marathon time points. Adjustments were performed by applying the method of Dill and Costill (1974), which uses hematocrit and hemoglobin to determine the magnitude of plasma volume changes after the race in each participant (Alis et al., 2015).

#### Urine Samples

Three urine samples (at the marathon day prior to run, at the finish line and at 48 h post-race) were also collected per participant to evaluate hematuria and hydration status. Samples were taken by the participant himself using sterilized recipients. Except at the finish line, participants were informed to collect the first-morning-void urine sample. The presence of blood in urine was firstly tested by using a dipstick (Aution Sticks 10EA, Arkray, Shiga, Japan), and only positive cases were then explored at microscopic level in order to count the number of red blood cells per field. Hematuria was considered when more than five erythrocytes per field were found in the urine sample (Grossfeld et al., 2001; Loo et al., 2009). Given that no morphological evaluation was performed, we could not determine the tissue origin of erythrocytes in the urine sediment. The urine’s specific gravity (USG; sediMax conTrust, 77 Elektonika Kft, Budapest, Hungary) was used to calculate hydration status (Casa et al., 2000; Kavouras, 2002).

#### Body Mass

For evaluating dehydration, we calculated the percentage of differences between the body mass before and after the completion of the marathon race (Noakes et al., 2005; Hoffman et al., 2017). Body mass was measured using a calibrated electronic scales (Seca 813, Vogel and Halke, Hamburg, Germany) on a firm surface, and runners wore their running clothes and shoes. Participants were not allowed to take a large meal 4 h prior to the prerace evaluation. At the finish line, participants were allowed to drink but not eat before measuring their body mass.

### Assessment of Renal Function

Acute kidney injury was evaluated according to the acute kidney injury network (AKIN) criteria (Mehta et al., 2007). Grades of AKI were defined as previously (González et al., 2019). The estimation of AKIN grades required the measurement of serum creatinine (sCr) levels and the GFR (Mehta et al., 2007; McCullough et al., 2011; Hodgson et al., 2017; Rojas-Valverde et al., 2021b).

Although sCr is the classic biomarker for monitoring renal function in healthy individuals at baseline (Rojas-Valverde et al., 2021b), we are aware that its kinetics can be influenced by the acute phase of muscle damage after long-distance running (Hodgson et al., 2017). However, we are specifically interested in its values through the recovery phase where muscle damage is limited (Bernat-Adell et al., 2019).

The GFR was estimated using the equation defined by The Chronic Kidney Disease Epidemiology Collaboration (CKD-EPI; Levey et al., 2009). We also calculated the relative increase of GFR at each time point with respect to their basal level (ΔGFR), by applying the following equation: fold increase (Δ) = (post-race value – pre-race value)/pre-race value.

We assessed the evolution of renal function through the entire study in each intervention group. In addition, we also compared the renal function between intervention groups in the intervention phase of the study to evaluate the impact of each recovery strategy on normalizing the different parameters measured.

### Statistical Analyses

Statistical analyses were carried out using the SPSS software v27, and two-sided values of *p* < 0.05 were considered statistically significant. The Kolgomorov-Smirnov test was used for testing data normality. Since variables were not normally distributed, non-parametric statistical tests were applied. To describe data collected, we used median and interquartile range (IQR) for continuous variables, and sample size and frequency (%) for categorical variables. The Friedman test was used to analyze the evolution of parameters over time in each intervention group. The Kruskal Wallis test was used for comparing parameters among groups at the post-intervention moments. Pairwise comparisons were performed using the Bonferroni method. Chi-square test was used for comparison of categorical variables among groups. ANOVA Levene test was used for comparison of quantitative variables among groups.

## Results

### Assessment of Renal Function Prior to Intervention

Race conditions (15.6°C on average temperature, 50% of humidity, and relatively short and flat race with non-significant elevation changes) limited the impact of well-known factors influencing AKI levels (Junglee et al., 2013; Hou et al., 2015; Rojas-Valverde et al., 2019, 2021a). In general, runnewrs were not dehydrated at the finish line (USG < 1.02 g/ml; Casa et al., 2000; Kavouras, 2002) and percentage of body loss was estimated around 3% (Noakes et al., 2005; Hoffman et al., 2017 in all groups; Supplementary Table S1). No significant differences in marathon performance were observed across AKI grades (Kruskal Wallis test, *p* = 0.262). No significant differences were observed between the three subsets of runners prior to intervention (Tables 1 and 2; Supplementary Table S1). Thus, we considered them to be sufficiently homogeneous for comparison.

**Table 1:** Description of study cohort.

Variable		RUN *N* = 22 (four females)	ELLIPTICAL *N* = 22 (four females)	REST *N* = 32 (six females)
Physiological characteristics^*^	Age	38.73 ± 3.92	37.86 ± 3.72	38.94 ± 3.26
	BMI	22.71 ± 1.27	23.49 ± 2.05	22.73 ± 1.74
	% body fat	14.74 ± 3.25	13.81 ± 3.67	19.54 ± 4.16
	Weight	67.88 ± 7.87	72.77 ± 10.86	71.03 ± 8.93
	Height	171.32 ± 8.47	173.73 ± 9.75	174.91 ± 7.84
	V·O_2max_ (ml·kg^−1^·min^−1^)	55.08 ± 6.04	53.96 ± 5.42	54.06 ± 6.21
	VT1 (ml·kg^−1^·min^−1^)	38.75 ± 3.80	38.48 ± 4.71	37.11 ± 4.45
	VT2 (ml·kg^−1^·min^−1^)	46.54 ± 4.35	45.87 ± 4.91	45.06 ± 4.49
Training indicators^*^	Sessions per week	4.73 ± 1.08	5.05 ± 0.67	4.84 ± 0.81
	Kilometers per week	61.82 ± 14.27	63.33 ± 11.33	65.16 ± 12.21
	Hours per week	7.00 ± 2.74	7.83 ± 2.90	7.36 ± 2.00
History as marathoner^*^	Marathons finished	3.41 ± 2.94	3.29 ± 3.02	2.69 ± 2.40
	Marathon per year	1.36 ± 0.90	1.14 ± 0.48	0.91 ± 0.39
Work intensity^#^	High intensity	4.50%	13.60%	6.30%
	Medium intensity	40.90%	18.20%	34.40%
	Low intensity	54.50%	68.20%	59.40%
Levels of study^#^	School graduate	4.50%	4.80%	6.30%
	High school graduate	4.50%	4.80%	9.40%
	Professional certificate	13.60%	23.80%	15.60%
	Undergraduate degree	77.30%	66.70%	68.80%

*N*, number of samples; F, female; BMI, body mass index; SD, standard deviation; *V·*O_2max_, maximal oxygen consumption; and VT, ventilatory threshold.

* Values are presented as mean ± SD.

# Values are presented as percentage of all individuals.

**Table 2:** Comparison of data collected prior to intervention.

Variable	RUN *N* = 22 (four females)	ELLIPTICAL *N* = 22 (four females)	REST *N* = 32 (six females)	*p*
Marathon time (min)	213.59 ± 20.24	216.40 ± 19.63	215.85 ± 21.87	0.869^*^
Absence AKI at the finish line	11 (50%)	9 (40.9%)	17 (53.1%)	0.341^#^
Presence AKI at the finish line	11 (50%)	11 (50%)	15 (46.9%)	
Grade 1 at the finish line	10 (45.5%)	10 (45.5%)	15 (46.9%)	
Grade 2 at the finish line	1 (4.5%)	1 (4.5%)	0 (0.0%)	
Presence of hematuria at the finish line	9 (40.9%)	8 (36.4%)	11 (34.4%)	0.886^#^
Presence of hematuria 48 h after marathon	0 (0%)	1 (4.5%)	2 (6.3%)	0.503^#^

*N*, number of subjects; F, female; AKI, acute kidney injury; and *p*, *p*-value. Data is presented as mean ± SD for continuous variables, and sample size (percentage) for categorical variables.

* ANOVA Levene test.

# Chi Square test.

**Figure 1:**
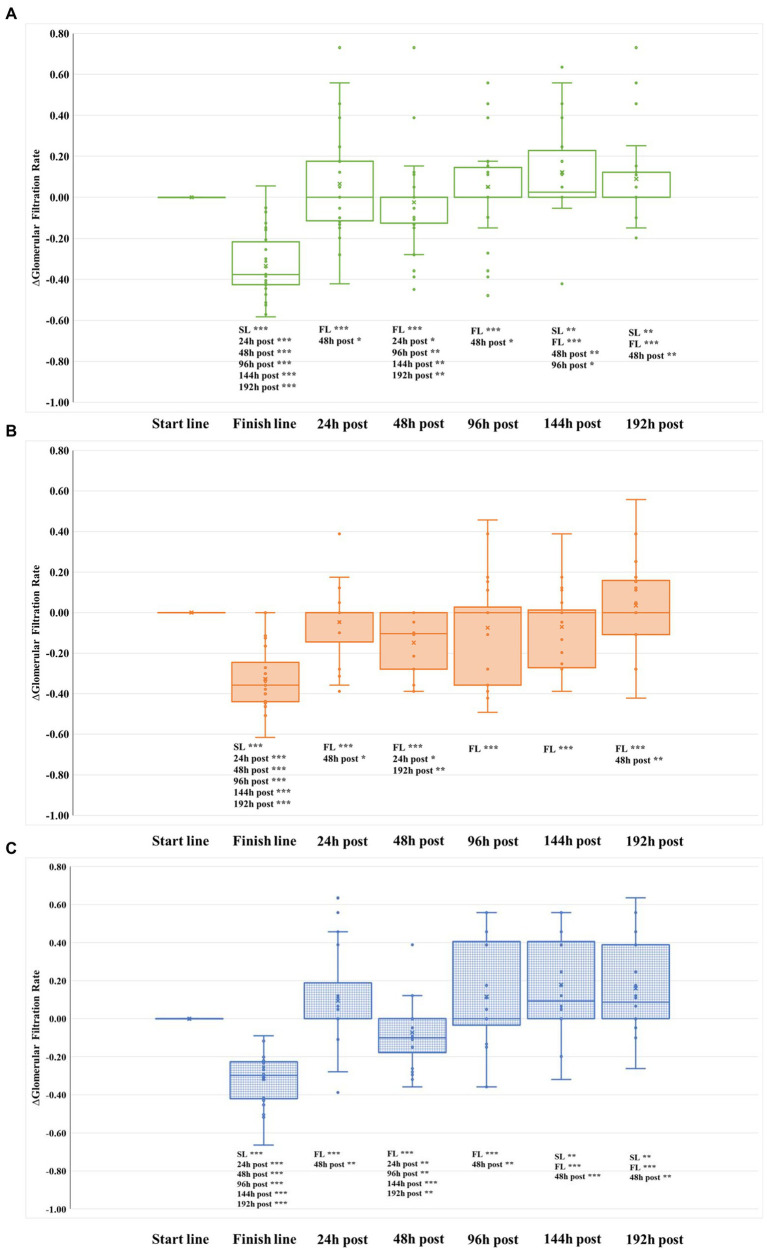
Evolution of glomerular filtration rate relative to baseline (∆GFR) in the three groups during the whole study. **(A)** REST group. **(B)** ELLIPTICAL group. **(C)** RUNNING group. SL, start line; and FL, finish line.

Kidney damage is normally observed after performing a highly demanding physical activity, such as running a marathon (McCullough et al., 2011; Traiperm et al., 2016; Mansour et al., 2017; González et al., 2019). According to levels of sCr and GFR collected at the finish line, 37 runners (48.68%) presented AKI immediately after running the marathon, being Grade I in 97% of cases and Grade II in the remaining 3% of cases (Table 2). The frequency of runners with kidney damage was similar across groups (*p* = 0.341). Biomarkers related to renal function (sCr, GFR, and ∆GFR) progressed similarly over time in the three groups (Table 3; Figure 1). All runners normalized the levels of these three biomarkers, and thus recovered from AKI, 24 h after finishing the marathon. However, an alteration of these parameters (increase of sCr and decrease of GFR and ∆GFR levels) was observed again at the 48 h post-marathon (Table 3; Figure 1). Our results are in concordance with previous studies (Irving et al., 1986, 1990; González et al., 2019).

**Table 3:** Evolution of serum creatinine (sCr) and the glomerular filtration rate (GFR) in the three groups during the whole study.

	Start line (1st time point)	Finish Line (2nd time point)	24 h post (3rd time point)	48 h post (4th time point)	96 h post (5th time point)	144 h post (6th time point)	192 h post (7th time point)	Friedmann *p* value
Serum creatinine (mg/dl)								
RUN *N* = 22 (four females)	1.00 [0.88–1.10]^2,6^	1.34 [1.15–1.49]^1,3,4,5,6,7^	0.90 [0,80-1,03]^2,4^	1.00 [0.90–1.10]^2,3,5,6,7^	0.90 [0.80–1.03]^2,4^	0.90 [0.80–1.00]^1,2,4^	0.90 [0.80–1.00]^2,4^	**3.80 × 10** ^**−14**^
^$^ELIPTICAL *N* = 22 (four females)	0.90 [0.80–1.00]^2,4^	1.26 [1.15–1.36]^1,3,4,5,6,7^	0.90 [0.85–1.00]^2,4^	1.00 [0,90–1.10]^1,2,3,7^	1.00 [0.80–1.10]^2^	0.90 [0.88–1.10]^2^	0.90 [0.80–1.00]^2,4^	**3.56 × 10** ^**−9**^
REST *N* = 32 (six females)	0.90 [0.80–1.08]^2,6^	1.31 [1.13–1.44]^1,3,4,5,6,7^	0.90 [0.80–1.00]^2^	1.00 [0.90–1.10]^2,6,7^	0.90 [0.80–1.08]^2^	0.90 [0.80–1.00]^1,2,4^	0.90 [0.80–1.00]^2,4^	**0.00**
Kruskal-Wallis *p* value	0.448	0.712	0.832	0.526	0.534	0.183	0.996	
Glomerular filtration rate (ml/min/1.73 m^2^)								
RUN *N* = 22 (four females)	72.91 [66.24–99.25]^2,6^	51.69 [44.17–61.03]^1,3,4,5,6,7^	94.64 [67.95–104.22]^2,4^	69.91 [62.95–90.27]^2,3,5,6,7^	95.69 [68.06–102.92]^2,4^	100,46 [73.30–105.26]^1,2,4^	98.06 [73.30–104.44]^2,4^	**1.10 × 10** ^**−13**^
^$^ELIPTICAL *N* = 22 (four females)	94.92 [71.65–100.50]^2,4^	54.60 [48.93–61.16]^1,3,4,5,6,7^	81.37 [68.93–98.76]^2,4^	69.41 [63.84–92.19]^1,2,3^	69.90 [61.53–101.20]^2^	80.52 [63.84–101.74]^2^	97.03 [69.17–105.12]^2^	**1.42 × 10** ^**−9**^
REST *N* = 32 (six females)	85.87 [66.54–97.88]^2,6^	51.10 [45.85–61.74]^1,3,4,5,6,7^	94.62 [69.29–102.74]^2^	71.90 [63.62–97.06]^2,6,7^	95.25 [64.72–100.62]^2^	97.37 [72.53–104.01]^1,2,4^	95.58 [72.15–102.02]^2,4^	**0.00**
Kruskal-Wallis *p* value	0.415	0.704	0.759	0.636	0.514	0.168	0.695	

Data is presented as median and interquartile range (IQR). Values of blood biomarkers in finish line (FL) were adjusted according to the method of Dill and Costill (1974). GFR was calculated using the Chronic Kidney Disease Epidemiology Collaboration (CKD-EPI) equation (Levey et al., 2009). *N*, number of participants; F, female; and *p*, *p*-value.

1,2,3,4,5,6,7 Significant differences between the different time points where data was collected after applying Bonferroni correction method. Bold font indicates statistical significance across all time points after applying Bonferroni correction method.

$ Blood samples from two participants (one female) included in the ELLIPTICAL group were not collected in the FL (2nd time point) because of logistic problems.

Hematuria was observed in urine samples collected from 28 runners (36.85%) at the finish line. No differences were observed in the frequency of runners with hematuria across subsets (*p* = 0.886). Except for two runners included in the REST group (6.2%) and one runner included in the ELLIPTICAL group (4.5%), hematuria disappeared in the urine samples collected 48 h after finishing the marathon.

### Impact of Recovery Strategy on Renal Function Normalization

We then explored the effect of three different workouts on kidney damage recovery following the completion of a marathon by monitoring three different biomarkers.

**Figure 2:**
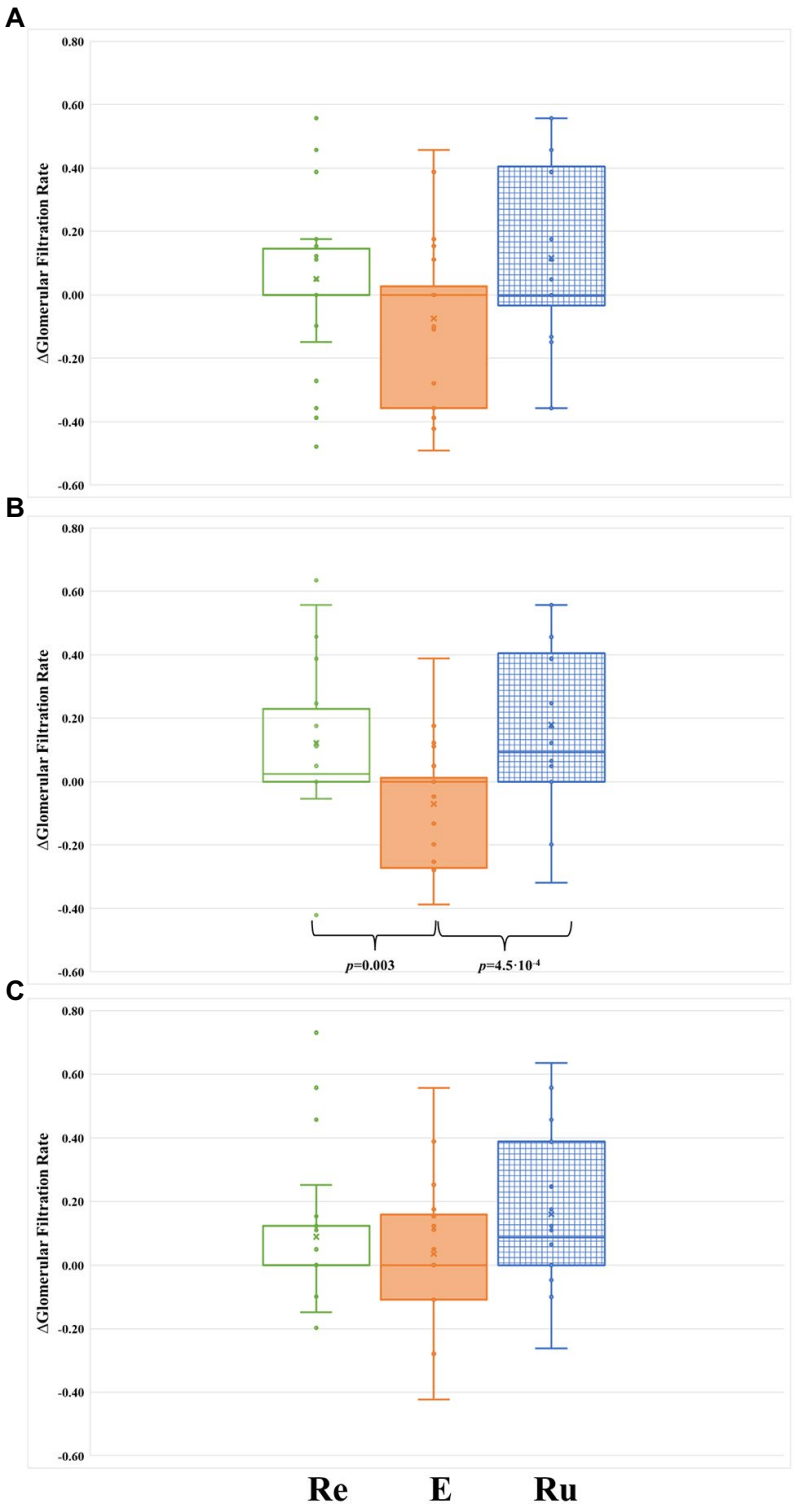
Comparison of ∆GFR between the tree groups during the intervention phase of the study. **(A)** After 48 h from the first intervention (at 96 h post-marathon). **(B)** After 48 h from the second intervention (at 144 h post-marathon). **(C)** After 48 h from the third intervention (at 192 h post-marathon). Re, participants included in the REST group (green-rimmed boxes); E, participants included in the ELLIPTICAL group (orange-filled boxes); and Ru, participants included in the RUN group (blue-dashed boxes).

From the first intervention (performed 96 h after finishing the marathon), the evolution of both sCr and GFR were similar in the three intervention groups (Table 3). However, we observed significant differences in the evolution of ∆GFR over time among groups (Figures 1, 2). Both RUN and REST groups normalized ∆GFR values 96 h after finishing the marathon, having a significantly better filtration rate than the basal level at the two last time points measured (144 and 192 h after finishing the marathon). Therefore, both resting and running seem to be convenient strategies for recovering from acute kidney injury.

Conversely, except at the 192 h post-marathon time point, the ELLIPTICAL group presented a lower relative filtration rate in all time points measured during the study (Figure 1B). Contrary to what we observed in the other two intervention groups, the ELLIPTICAL group did not show significantly better filtration rates after finishing the recovery phase of the study. We are aware that the ELLIPTICAL group did not show improvement in filtration rates 24 h after finishing the marathon with respect to baseline, and this group also showed the lowest ∆GFR values prior to intervention (48 h post-marathon). However, the lack of significant differences in filtration rates observed prior to intervention between groups supports that such workout is not optimal for renal damage recovery.

Finally, we compared the ∆GFR levels between groups at each time point of the intervention phase (Figure 2). Significant differences in ∆GFR were only observed in samples collected immediately before starting the second recovery workout (144 h post-marathon: *p* = 0.001). Specifically, the ELLIPTICAL group showed a significantly lower ∆GFR compared to both the RUN group (*p* = 4.5 × 10^−4^; Figure 2B) and the REST group (*p* = 0.003; Figure 2B).

**Figure 3:**
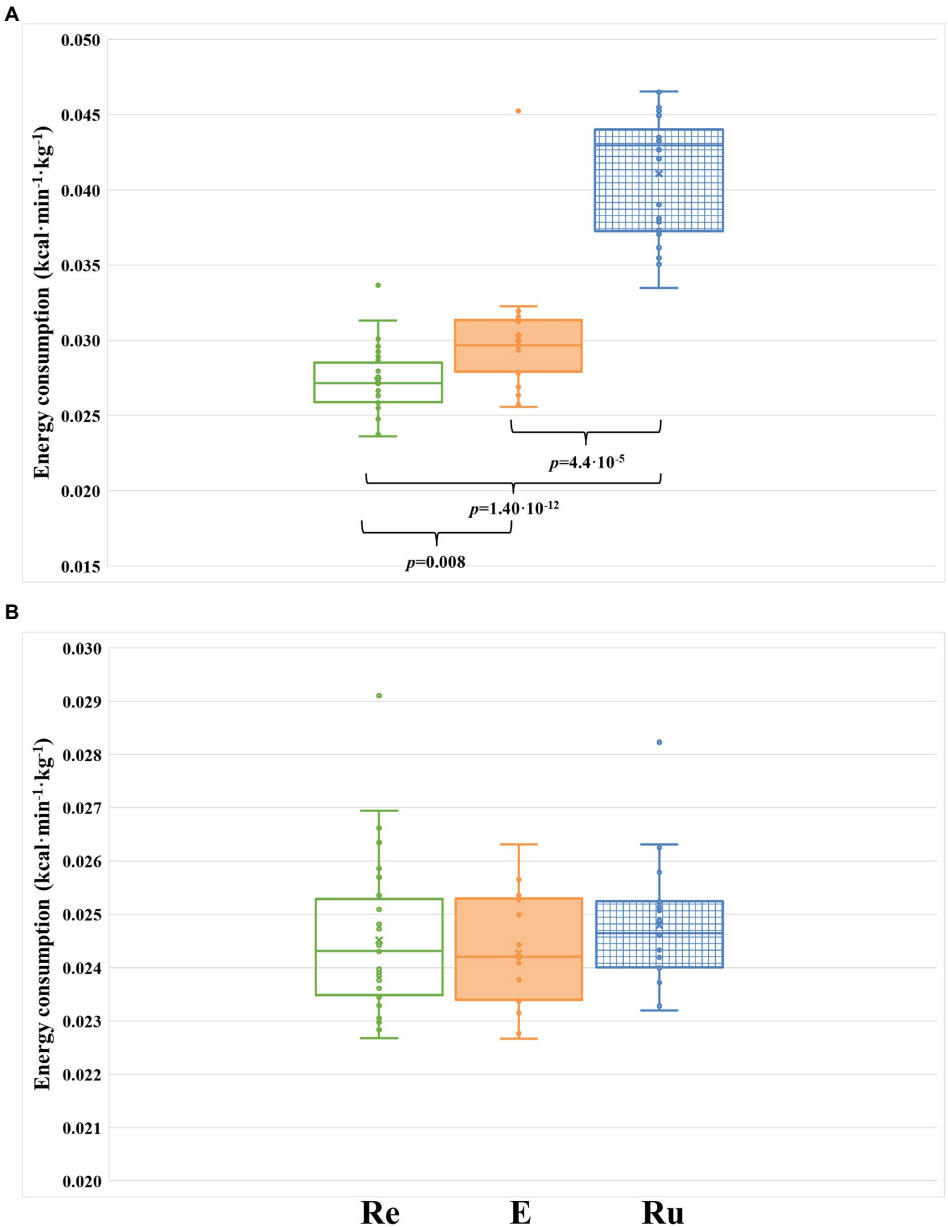
Comparison of energy consumption (kcal/kg/min) between the three groups during the intervention phase of the study. **(A)** Energy consumed by each group during the three 8-h segments where the recovery activity was performed (24 h in total). **(B)** Energy consumed by each group during the time of the intervention phase where runners are not performing the activity (13 8-h segments). Re, participants included in the REST group (green-rimmed boxes); E, participants included in the ELLIPTICAL group (orange-filled boxes); and Ru, participants included in the RUN group (blue-dashed boxes).

To control that runners strictly followed the activity proposed, we measured the caloric consumption of each participant through the entire intervention phase of the study. No differences in caloric consumption were found between the three groups when they were not performing the recovery activity (*p* = 0.354; Figure 3B), confirming that runners did not perform any extra physical exercise apart from the one controlled by us. However, when only the time of the recovery activity was considered, we observed highly significant differences in the caloric consumption between groups (*p* = 1.22 × 10^−11^; Figure 3A). As expected, the REST group consumed significantly less calories than the RUN group (*p* = 1.40 × 10^−12^) and the ELLIPTICAL group (*p* = 0.008). We also observed that the RUN group consumed significantly more calories than the ELLIPTICAL group (*p* = 4.4 × 10^−5^). Thus, running seems to be the physical activity that requires the highest caloric consumption (Figure 3A).

## Discussion

Here, we present a robust study focused on exploring acute kidney failure and its recovery after running a marathon, one of the most physically demanding activities. As far as we are aware, this is the first interventional study testing three strategies for accelerating AKI recovery following a strenuous exercise. Our approach not only complements previous studies looking for optimal recovery strategies (Kellmann et al., 2018; Wiewelhove et al., 2018; Martínez-Navarro et al., 2020b; Kwiecien et al., 2021), but also presents a novel experimental design to continuously monitor participants through the whole study, limiting thus the uncontrolled factors that may influence results. In addition, unlike previous studies (Sherman et al., 1984), the intensity of the recovery activity was defined and thus not selected by the individual him/herself so that all participants performed workouts at an equivalent relative intensity. Our approach, together with the substantial number of participants included in the study, allowed us to comprehensively define the best strategy for recovering from acute renal injury.

To monitor renal function, we decided to measure levels of a classical renal function biomarker (sCr). This biomarker is well established for monitoring renal function in healthy individuals, but can be influenced by acute muscle damage (Hodgson et al., 2017). As previously shown (Irving et al., 1986; González et al., 2019), the depression of GFR seems to be biphasic. This observation could be influenced by the evolution of muscle damage biomarkers – a similar recovery pattern of a well-known muscle damage biomarker (LDH) have been previously described (Bernat-Adell et al., 2019). Therefore, analyzing novel biomarkers [i.e., neutro-phil gelatinase-associated lipocalin (NGAL), kidney injury molecule-1 (KIM-1), or Cystatin (C)] would be relevant to assess acute kidney damage caused by high-intensity physical activities in order to avoid their overestimation (McCullough et al., 2011; Hodgson et al., 2017). In addition, these new biomarkers reflect intracellular alterations being more sensitive and specific to evaluate acute glomerular and tubular damage (Panizo et al., 2015). However, these novel biomarkers are still not validated for long-term renal function evaluation and they are not cost-effective diagnostic markers (Poortmans et al., 2013; Hodgson et al., 2017; Rojas-Valverde et al., 2021b). In addition, we measured renal function at the recovery phase where muscle damage is limited (Rojas-Valverde et al., 2021b). Further work is required to explore renal function recovery after strenuous exercise using novel biomarkers.

The biphasic depression of GFR observed in our study, apart from being correlated with muscle damage levels (Bernat-Adell et al., 2019), could also be related with the hydration status (Poortmans, 1984; Poortmans et al., 1988). Runners tend to increase their fluid intake after marathon running, which may promote glomerular filtration; but it usually backs to normal after 48 h post-marathon running. Similar adequate hydration levels were observed 48 h after completing the marathon race compared to those measured at the finish line. The observed transient GFR recovery at the 24 h post-marathon time point may be due to overhydration. However, since there is no information about both the hydration status at this time point and the rehydration strategy followed by runners, this hypothesis should be tested and validated in future studies.

Previous studies in the field have generally focused on measuring biomarkers of muscle and cardiovascular damage. Up to now, only two studies monitored renal function for more than 48 h after finishing a long distance race to evaluate how long of full resting is needed for renal function recovery (Irving et al., 1986, 1990). According to their results, levels of serum creatinine remained significantly elevated up to 72 h post-race. The inconsistency with our observations may be due to the limited sample size of these studies (less than 10 individuals analyzed) and the daily activities performed by participants during the recovery period (they were not continuously monitored as in our study).

Recently, in the same study population, we reported data on muscle damage recovery after running a marathon (Martínez-Navarro et al., 2020b). We observed that both active and passive recovery had similar effects in muscle damage recovery, which was supported by a previous study (Sherman et al., 1984). However, the RUN group showed a faster recovery in neuromuscular function compared to REST and ELLIPTICAL groups. Therefore, we concluded that running at 95–100% of VT1 seemed to be the optimal strategy for muscle function recovery 48 h after finishing the marathon, as long as pain did not prevent exercise from being properly performed. In case of muscle pain, we recommended runners to perform elliptical workouts during the week after marathon racing (Martínez-Navarro et al., 2020b).

However, results achieved in this study lead us to strongly advise against the use of elliptical machines for marathon recovery because of its negative impact on renal function recovery. Hence, we encourage runners to carry out an active recovery based on light-intensity continuous running from 48 h after finishing the marathon. This will promote both muscular and renal function recovery. Our results also suggest that full resting is a better strategy than using an elliptical machine (its use should be delayed until at least 1 week after marathon running). This observation is contrary to our initial hypothesis, and further work is also required to understand why runners who perform elliptical workouts (a lower-impact exercise for lower-limb joints) recovered later from renal damage. Note that our observations were not affected by the dehydration status, which has been shown to limit renal recovery (Poortmans, 1984; Panizo et al., 2015; Rojas-Valverde et al., 2021b), since all participants had a correct hydration prior to the recovery phase.

The main weakness of our study is the need of selecting comparable recovery strategies in terms of biomechanical movements for being able to monitor participants using triaxial accelerometers. This limitation is caused by the lack of validation of accelerometer activity-level specific cut-offs for cycling and swimming in a cohort with a substantial level of physical activity compared to normal population. Moreover, being able to swim during 40 min at 95–105% VT1 requires a previous adaptation impeding to randomly include participants in an intervention group using swimming as recovery strategy. The fact that the elliptical machine is not normally used by participants for training can also be a limitation, since it may be uncomfortable and hard to coordinate leg and arm movements. In addition, it is not well-known whether elliptical workout requires a greater physical effort at the muscular (not joint) level compared to running, which may in fact delay renal recovery. As discussed above, another limitation of our study is the impact of muscle damage on classical renal injury biomarkers.

In summary, our results show the beneficial impact of light-intensity continuous running on marathon-induced physiological damage recovery. Our study is an important resource to guide runners, coaches, and medical specialists in their search for the most optimal recovery strategy after running a marathon.

## Data Availability Statement

The original contributions presented in the study are included in the article/**Supplementary Material**, further inquiries can be directed to the corresponding author.

## Ethics Statement

The studies involving human participants were reviewed and approved by Research Ethics Committee of the Jaume I University of Castellon. The patients/participants provided their written informed consent to participate in this study.

## Author Contributions

CarlosH and BH contributed to conception and design of the study, article drafting, and critical revision of the article. CarlosH and CarlaH contributed to data curation, analysis, and interpretation. CarlosH, IM-N, EC-B, AF-A, and NP contributed to data collection and critical revision of the article. CarlosH, IM-N, and EC-B contributed to funding acquisition. All authors contributed to the article and approved the submitted version.

## Funding

The logistics of the study was funded by Fundacion Trinidad Alfonso. All cardiovascular, blood, and urine tests were funded by Vithas-Nisa Hospitals group. The funding resource was received from Sociedad Deportiva Correcaminos.

## Conflict of Interest

The authors declare that the research was conducted in the absence of any commercial or financial relationships that could be construed as a potential conflict of interest.

## Publisher’s Note

All claims expressed in this article are solely those of the authors and do not necessarily represent those of their affiliated organizations, or those of the publisher, the editors and the reviewers. Any product that may be evaluated in this article, or claim that may be made by its manufacturer, is not guaranteed or endorsed by the publisher.

## Footnotes

^1^ www.clinicaltrails.gov
