# Supplementary material for: Renal Function Recovery Strategies Following Marathon in Amateur Runners
Source: Front Physiol. 2022 Feb 28;13:812237. doi: 10.3389/fphys.2022.812237 (PMC8918951; doi:10.3389/fphys.2022.812237)
Supplement: Supplementary file 1 [file Table_1.pdf]

Table S1. Evaluation of hydration status groups during the whole study					
	**Urine specific gravity (g/ml)**				**Percentage of body mass loss at the finish line**
	Start Line (1st time point)	Finish Line (2nd time point)	48h post (4th time point)	Friedmann *p* value	
RUN N=22 (4F)	1.019 [1.016-1.022][a]	1.019 [1.012-1.024]	1.015 [1.009-1.022][a]	**0.046**	3.00 [2.00-4.00]
ELIPTICAL N=22 (4F)	1.019 [1.016-1.021]	1.019 [1.015-1.024]	1.017 [1.011-1.021]	0.279	3.00 [2.00-4.00]
REST N=32 (6F)	1.018 [1.014-1.021]	1.016 [1.012-1.022]	1.015 [1.010-1.022]	0.343	3.00 [2.00-3.75]
Kruskal-Wallis *p* value	0.661	0.392	0.835		0.523

Data is presented as median and interquartile range [IQ].

Abbreviations: N, number of participants; F, female; *p*, p value.

[a]Significant differences between the different time points where data was collected after applying Bonferroni correction method
